# Efficacy and safety of Chinese herbal compound in the treatment of functional constipation: A protocol for systematic review and meta-analysis

**DOI:** 10.1097/MD.0000000000022456

**Published:** 2020-09-25

**Authors:** Lijiang Ji, Yihua Fan, Linhui Li, Huiwen Bai, Liping Weng, Ping Zhao

**Affiliations:** a Department of Anorectal Surgery, Changshu Hospital Affiliated to Nanjing University of Chinese Medicine, Changshu, Jiangsu province; b First Teaching Hospital of Tianjin University of Traditional Chinese Medicine; c Tianjin University of Traditional Chinese Medicine, Tianjin, China.

**Keywords:** bowel function, Chinese herbal compound, functional constipation, protocol, systematic review

## Abstract

**Background:**

Functional constipation refers to constipation without organic lesions caused by dietary factors, mood depression, changes in life rules, and poor bowel habits. Clinically, constipation is mainly manifested by changes of stool texture, difficulty or lack of bowel movement, and dry stool. Sometimes, it can be accompanied by abdominal distension and abdominal discomfort. Chinese herbal compound is a prescription which is composed of 2 or more medicinal flavors and is designed for relatively certain diseases and syndromes. Clinical studies have shown that TCM compounds have a good therapeutic effect on functional constipation, but there is no evidence of evidence-based medicine. The purpose of this study is to systematically evaluate the efficacy and safety of TCM compounds in the treatment of functional constipation, and to provide evidence-based basis for the clinical application of TCM compounds in the treatment of constipation.

**Methods:**

A systematic search was performed on English database (PubMed, Embase, Web of Science, the Cochrane Library) and Chinese database (CNKI, Wanfang, Weipu (VIP), CBM), in addition to the manual retrieval of Baidu and Google academic for randomized controlled trials (RCTs) on the treatment of functional constipation with Chinese herbal compound. The retrieval time limit was from the establishment of the database to August 2020. Two researchers independently screened the literature, extracted the data and evaluated the quality of the included studies. A meta-analysis was performed using RevMan5.3 software.

**Results:**

In this study, the efficacy and safety of TCM herbal compounds in the treatment of functional constipation were evaluated by the overall effective rate, recovery rate, colonic transmission function, and other indicators.

**Conclusions:**

This study will provide reliable evidence-based evidence for the clinical application of Chinese herbal compound in the treatment of functional constipation.

**OSF Registration number:**

DOI 10.17605/OSF.IO/D5ECF.

## 1 Introduction

Functional constipation (FC) is a common functional bowel disorder in clinical practice, manifesting as straining during defecation, lumpy or hard stools and infrequent bowel movements, in the absence of evident organic or structural diseases.^[1]^ Functional constipation can not only cause hemorrhoids, anal fissure, colon cancer, and other disease syndromes, but also induce and aggravate cardiovascular and cerebrovascular diseases, leading to acute myocardial infarction, cerebrovascular accident, and other diseases, endangering life.^[2]^ Existing studies have shown that the prevalence of functional constipation seems to increase gradually in the age of 50, and reaches the maximum after the age of 70, and the prevalence of functional constipation is generally higher in females than in males.^[3]^ The etiology of functional constipation is very complex, including: abnormal functions of colon, rectum, anal canal and pelvic floor muscles,^[4]^ intestinal flora imbalance,^[5]^ mental depression and psychological anxiety,^[6]^ smoking and alcohol abuse,^[7]^ poor bowel habits, poor dietary habits^[8]^ and dietary structure,^[9]^ gender and age, and genetic factors. In clinical practice, western medicine mainly treats functional constipation by adjusting diet structure, biofeedback therapy,^[10]^ drugs, surgery, etc.^[11]^

Traditional Chinese medicine compound is composed of 2 or more herbs, which is one of the main intervention methods in TCM clinic. Based on the theory of traditional Chinese medicine, the compound medicine addresses both symptoms and root causes by adjusting the zang-fu organs, Yin and Yang, Qi and blood, and using the medicine according to syndrome differentiation. Rich experience has been accumulated in the treatment of constipation with traditional Chinese medicine, which can provide individualized treatment programs according to different conditions of patients to restore intestinal function, with safety and effectiveness, few adverse reactions and no drug dependence.^[12]^

At present, although many clinical studies have shown that Traditional Chinese medicine has a significant effect on functional constipation, with high cure rate, low recurrence rate, and few adverse reactions, however, the number of clinical trials is small, and there are differences in research design and efficacy, which to some extent affect the promotion of this therapy.

Therefore, in this study, we conducted a meta-analysis to investigate the effects of TCM compounds on bowel function and quality of life in patients with functional constipation, so as to provide a reliable evidence-based basis for the treatment of functional constipation by TCM compounds.

## 2 Methods

### 2.1 Protocol register

This protocol of systematic review and meta-analysis has been drafted under the guidance of the preferred reporting items for systematic reviews and meta-analyses protocols (PRISMA-P). Moreover, it has been registered on open science framework (OSF) on August 28, 2020. (Registration number: DOI 10.17605/OSF.IO/D5ECF).

### 2.2 Ethics

Since this is a protocol with no patient recruitment and personal information collection, the approval of the ethics committee is not required.

### 2.3 Eligibility criteria

#### 2.3.1 Types of studies

We will collected all available randomized controlled trails(RCTs) on Chinese herbal compound treatment for functional constipation, regardless of blinding, publication status, region, but Language will be restricted to Chinese and English.

#### 2.3.2 Types of participants

1. In accordance with the diagnostic criteria of Rome(II, III, IV) for functional constipation;
2. No restriction on age, race or gender;
3. No other complications.

#### 2.3.3 Types of interventions

The treatment group: use traditional Chinese medicine compound intervention (decoction, Chinese patent medicine) only or combined with western medicine;

The control group: western medicine intervention or placebo.

#### 2.3.4 Types of outcome measures

1. Main outcome: Overall effective rate, recovery rate and colonic transmission function;
2. Secondary outcome: Symptom improvement, recurrence rate, quality of life score, and intestinal flora level.

### 2.4 Exclusion criteria

1. Studies published repeatedly;
2. Studies which were abstracts and conference papers, and in which the original data cannot be obtained;
3. Study whose data was incomplete or where there were obvious errors that cannot be handled after contacting the author;
4. Study with wrong random method;
5. Organic constipation or constipation due to other secondary causes;
6. Intervention measures other than traditional Chinese medicine compound or combined with other therapies (such as acupuncture, massage, qigong exercise, psychological therapy).

### 2.5 Search strategy

“Traditional Chinese medicine”(zhong yao), “functional constipation”(gong neng xing bian mi) and other Chinese search terms were used for retrieval in Chinese databases, including CNKI, Wanfang Data Knowledge Service Platform, VIP Information Chinese Journal Service Platform, and China Biomedical Database. English retrieval words such as “traditional Chinese medicine”, “Chinese patent medicine”, “Chinese medicine prescription”, “functional constipation” were used for retrieval in English databases, including PubMed, EMBASE, Web of Science and the Cochrane Library. In addition, manual retrieval was performed in Baidu and Google academic. The retrieval time was from the establishment of the database to August 2020, and all domestic and foreign literatures on the treatment of functional constipation with Traditional Chinese medicine were collected. Take PubMed as an example, and the retrieval strategy is shown in Table 1.

**Table 1:**
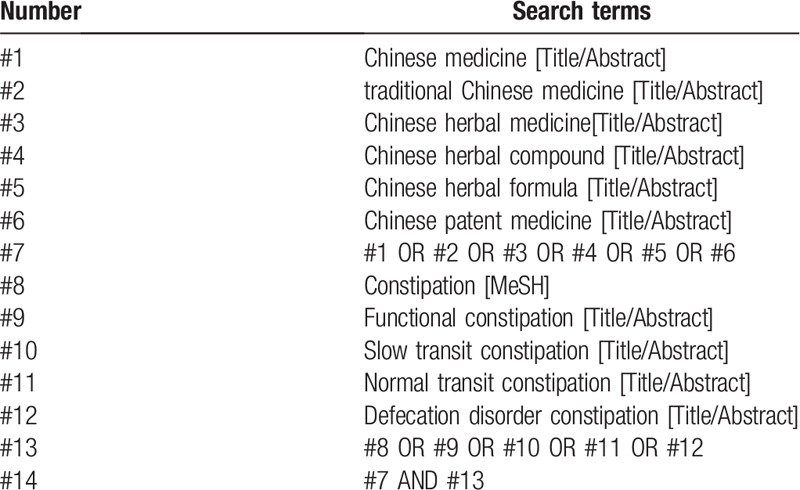
Search strategy in PubMed database.

### 2.6 Data screening and extraction

**Figure 1:**
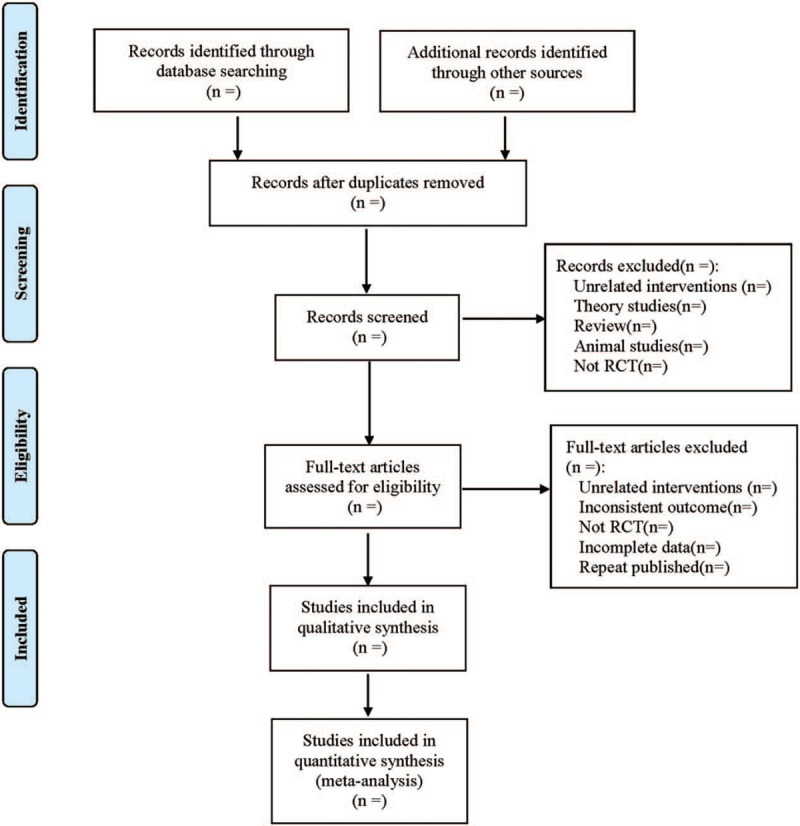
Flow diagram.

Cochrane Collaboration System Reviewer Manual Version 5.0 was used as a reference for the method of selection in the study. According to the PRISMA flow chart, EndNote X7 document management software was utilized by 2 researchers to independently screen the documents based on the above inclusion and exclusion criteria before mutual check. Those difficult to determine whether included in the study, would be discussed and judged with a third researcher. At the same time, Excel 2013 was used to extract relevant information, including: ① Clinical features (title, first author, publication year and month, sample size, sex ratio, average age, average course of disease); ② Intervention measures: The name, composition, dosage form, frequency and course of treatment of the Chinese herbal compound used in the treatment group, the name, frequency, and course of treatment of western medicine;

The name, administration method, frequency, and course of treatment of western medicine used in the control group; ③ Evaluation factors of risk bias in randomized controlled studies; ④ Observation indicators. The literature screening process is shown in Figure 1.

### 2.7 Literature quality evaluation

Built-in Risk bias evaluation tool of Review Manager 5.3 Software (the Cochrane collaborations tool for assessing risk of bias) was used to assess the risk bias in the included studies. Two researchers determined the literatures from 3 levels, including low-risk, unclear, and high-risk based on the performance of the included literature in the above evaluation items. After completion, they would recheck. In case of a disagreement, they would discuss. If no agreement could be reached, a decision would be made in consultation with researchers from the third party.

### 2.8 Statistical analysis

#### 2.8.1 Data analysis and processing

The RevMan 5.3 software provided by the Cochrane Collaboration was used for statistical analysis. ① For dichotomous variables, relative risk (RR) was used for statistics. For continuous variables, weighted mean difference (WMD) was selected when the tools and units of measurement indicators are the same, standardized mean difference (SMD) was selected with different tools or units of measurement, and all the above were represented by effect value and 95% Confidence interval (CI). ② Heterogeneity test: Q test was used to qualitatively determine inter-study heterogeneity. If *P* ≥ .1, there was no inter-study heterogeneity, If *P* < .1, it indicated inter-study heterogeneity. At the same time, *I*^2^ value was used to quantitatively evaluate the inter-study heterogeneity. If *I*^*2*^ ≤ 50%, the heterogeneity was considered to be good, and the fixed-effect model was adopted. If *I*^*2*^ > 50%, it was considered to have significant heterogeneity, the source of heterogeneity would be explored through subgroup analysis or sensitivity analysis. If there was no obvious clinical or methodological heterogeneity, it would be considered as statistical heterogeneity, and the random-effect model would be used for analysis. Descriptive analysis was used if there was significant clinical heterogeneity between the 2 groups and subgroup analysis was not available.

#### 2.8.2 Dealing with missing data

If data is missing or incomplete, we will contact the corresponding author to obtain the missing data. If not, this study will be removed.

#### 2.8.3 Subgroup analysis

Subgroup analysis was carried out according to the treatment group of Chinese herbal compound assisted other therapies and Chinese herbal compound treatment only; subgroup analysis was carried out according to the age of the patients, which can be divided into 4 subgroups: minors, young people, middle-aged people, and elderly people. Subgroup analysis was carried out according to the clinical classification of functional constipation, which can be divided into 3 subgroups: normal type, slow type, and defecation disorder type. Subgroup analysis was carried out according to the TCM syndromes, which can be divided into 5 subgroups: xu mi(deficient constipation), re mi(constipation due to heat), leng mi(constipation due to interior cold), qi mi(constipation due to qi stagnation), xue mi(constipation with blood deficiency syndrome). Subgroup analysis was carried out according to the types of Chinese herbal compound used.

#### 2.8.4 Sensitivity analysis

In order to test the stability of meta-analysis results of outcomes, a one-by-one elimination method will be adopted for sensitivity analysis.

#### 2.8.5 Assessment of reporting biases

For the major outcome indicators, if the included study was ≥10, funnel plot was used to qualitatively detect publication bias. Eggers and Beggs test are used to quantitatively assess potential publication bias.

#### 2.8.6 Evidence quality evaluation

The Grading of Recommendations Assessment, Development, and Evaluation (GRADE) will be used to assess the quality of evidence. It contains 5 domains (bias risk, consistency, directness, precision, and publication bias). And the quality of evidence will be rated as high, moderate, low, and very low.

## 3 Discussion

Functional constipation belongs to the categories of “constipation” (Bian mi) and “difficulty in defecation” (Da bian nan) in Traditional Chinese medicine. In the early stage of TCM, a lot of experience has been accumulated in the clinical treatment of functional constipation, and many effective therapies have been designed.^[13]^ According to traditional medicine, the etiology and pathogenesis of constipation can be divided into 5 aspects:

1. “Re mi”(constipation due to heat) caused by gastrointestinal heat and body fluid consumption.
2. “Qi mi”(constipation due to qi stagnation) caused by stagnation of the qi and abnormal ventilation and depression.
3. “Leng mi”(constipation due to interior cold) caused by excrescent Yin and cold stagnating in the gastrointestinal tract.
4. “Yang xu mi”(constipation with yang deficiency syndrome) caused by Qi and Yang deficiency, Yang qi cannot pushing and defecation time prolonging.
5. “Xue xu mi”(constipation with blood deficiency syndrome) caused by Yin and blood deficiency, which leads to defecation difficulties.^[14]^

Through treatment based on syndrome differentiation, TCM often uses tonifying, Catharsis, and warming methods in clinical treatment of functional constipation with different syndromes. Studies have shown that Buzhong Yiqi Decoction can regulate the secretion of gastrointestinal hormones, elevate the level of Human serum Substance P (SP), reduce the level of Plasma Motilin (MTL), enhance gastrointestinal motility, and improve gastrointestinal motility disorders.^[15]^ Both Xuanfei Tongbian Decoction and Maziren Pill could regulate the serum enteric neurotransmitter level by up-regulating SP and down-regulating nitric oxide (NO),^[16]^ improve gastrointestinal motility, and play a role in the treatment of functional constipation.^[17]^ Yiqi Kaimi Prescription and Qishen Gutuo Mixture can increase the anal resting pressure, restore the anorectal sensory function and the coordination of pelvic floor muscle groups,^[18]^ and thus restore the normal process of voluntary defecation.^[19]^ Wenyang Xuanfei Prescription can significantly improve the expression level of Cajal mesenchymal cell-specific protein C-Kit and Stem Cell Factor (SCF) in rat colon by tonifying kidney and warming Yang, shorten defecation time, and promote intestinal peristalsis.^[20]^ Xiaochaihu Decoction can relieve anxiety and depression symptoms of some patients with functional constipation, improve their defecation ability, and improve constipation.^[21]^ Yiqi Xuanfei Tongbian Prescription can regulate gastrointestinal flora level, inhibit the release of inflammatory factors, promote the release of gastrointestinal secretions, and improve gastrointestinal function.^[22]^ Sini Powder and Yunchang Capsule can shorten the colon transmission time and improve constipation symptoms.^[23,24]^ A variety of traditional Chinese medicine compound components can also regulate the expression of aquaporins (AQPs) in the intestine and maintain the balance of water and liquid metabolism in the body.^[25]^

At present, there are many widely reported trials on different TCM compounds for the treatment of functional constipation, and TCM compounds have achieved good clinical efficacy in the treatment of functional constipation,^[26]^ but there is a lack of systematic and correct evaluation. Therefore, it is necessary to objectively evaluate the effect of TCM compounds on patients with functional constipation through evidence-based medicine, promote the treatment of TCM, and provide scientific and evidence-based TCM prescriptions for clinical practice. However, this study also has some limitations. The composition, dosage and course of treatment of the Chinese herbal compound included in the study were inconsistent, there may be some clinical heterogeneity, and the follow-up time is short, so it is difficult to evaluate the long-term efficacy of the Chinese herbal compound. In addition, many studies did not involve allocation-hiding and blind methods, and the methodological quality was poor, with some bias. At the same time, due to the limitation of language ability, only English and Chinese literature were searched, and studies in other languages may be ignored, which may lead to certain publication bias.

## Author contributions

**Data curation:** Lijiang Ji, Yihua Fan.

**Funding acquisition:** Ping Zhao.

**Investigation:** Ping Zhao.

**Resources:** Linhui Li, Huiwen Bai.

**Software:** Linhui Li, Huiwen Bai, Liping Weng.

**Supervision:** Ping Zhao.

**Writing – original draft:** Lijiang Ji, Yihua Fan.

**Writing – review & editing:** Lijiang Ji.
